# Super resolution deep learning reconstruction for coronary CT angiography: A structured phantom study

**DOI:** 10.1016/j.ejro.2024.100570

**Published:** 2024-05-24

**Authors:** Toru Higaki, Fuminari Tatsugami, Mickaël Ohana, Yuko Nakamura, Ikuo Kawashita, Kazuo Awai

**Affiliations:** a Graduate School of Advanced Science and Engineering, Hiroshima University, Japan; b Graduate School of Biomedical and Health Sciences, Hiroshima University, Japan; c Dept. Radiology, University of Strasbourg, France

**Keywords:** Super-resolution deep-learning-based reconstruction, Structured phantom, Computed tomography, Image quality

## Abstract

**Purpose:**

Super-resolution deep-learning-based reconstruction: SR-DLR is a newly developed and clinically available deep-learning-based image reconstruction method that can improve the spatial resolution of CT images. The image quality of the output from non-linear image reconstructions, such as DLR, is known to vary depending on the structure of the object being scanned, and a simple phantom cannot explicitly evaluate the clinical performance of SR-DLR. This study aims to accurately investigate the quality of the images reconstructed by SR-DLR by utilizing a structured phantom that simulates the human anatomy in coronary CT angiography.

**Methods:**

The structural phantom had ribs and vertebrae made of plaster, a left ventricle filled with dilute contrast medium, a coronary artery with simulated stenosis, and an implanted stent graft. By scanning the structured phantom, we evaluated noise and spatial resolution on the images reconstructed with SR-DLR and conventional reconstructions.

**Results:**

The spatial resolution of SR-DLR was higher than conventional reconstructions; the 10 % modulation transfer function of hybrid IR (HIR), DLR, and SR-DLR were 0.792-, 0.976-, and 1.379 cycle/mm, respectively. At the same time, image noise was lowest (HIR: 21.1-, DLR: 19.0-, and SR-DLR: 13.1 HU). SR-DLR could accurately assess coronary artery stenosis and the lumen of the implanted stent graft.

**Conclusions:**

SR-DLR can obtain CT images with high spatial resolution and lower noise without special CT equipments, and will help diagnose coronary artery disease in CCTA and other CT examinations that require high spatial resolution.

## 1 Introduction

Coronary CT angiography (CCTA), which can noninvasively exclude coronary artery disease (CAD) [1], [2], [3], has been widely used due to the availability of multiple-row detector CT and radiation dose reduction with various image reconstruction methods. In CCTA, it is important to accurately visualize coronary arteries and other fine structures such as small calcified lesions and inserted stent grafts.

Ultra-high-resolution CT (UHR-CT) has been developed as a way to increase the spatial resolution of CT, and is now being used in clinical practice. UHR-CT has approximately twice the spatial resolution compared to conventional CT systems, and its applications in CCTA have been reported to improve the diagnostic performance of CAD [4], [5], [6]. In recent years, photon-counting detector CT (PCD-CT), which employs a semiconductor detector, has also come into clinical use as a CT device capable of high-resolution imaging. With PCD-CT, CCTA can be performed with high spatial resolution and is expected to improve the diagnostic accuracy of CAD [7], [8], [9]. However, all of these require special CT equipment and have therefore limited availability.

Super-resolution deep learning reconstruction (SR-DLR) has developed as another way to improve the spatial resolution of CT images [10]. It has been reported that conventional DLR has better noise reduction and can output diagnostically acceptable images even in low-dose scans [11]. The newly developed SR-DLR can improve the spatial resolution of images acquired with normal-resolution CT systems and output images similar to those acquired with a UHR-CT. Sato et al. investigated the image quality characteristics of SR-DLR using a simple phantom and reported that SR-DLR has higher spatial resolution and less image noise than conventional reconstruction methods [12]. Nagayama et al. and Tatsugami et al. applied SR-DLR to clinical cases and reported that it was visually and semi-quantitatively superior to conventional image reconstruction methods [13], [14].

Nonlinear image reconstruction methods such as DLR are known to vary in their properties depending on the structure of the scanned object [15], [16], and their properties cannot be fully evaluated on a phantom with a simple structure. In addition, quantitative image qualities such as spatial resolution are difficult to measure on clinical images. To solve these problems, this study investigates the quantitative image quality of SR-DLR using a structural phantom that simulates the human anatomy.

## 2 Materials and Methods

### 2.1 Super-resolution deep learning reconstruction

The SR-DLR (Precise IQ Engine: PIQE, Canon Medical Systems Corp., Japan) is an image reconstruction method based on deep learning that can output high spatial resolution images from data acquired by normal-resolution CT [10]. A deep convolutional neural network (DCNN), the core part of SR-DLR, was trained using UHR-CT images as a target to improve the spatial resolution of the input images. At the same time, the DCNN was trained on a noise-free image, which also reduces the noise in the input image. The SR-DLR is clinically available on clinical 320-row detector CT (Aquilion ONE, Canon Medical Systems Corp., Japan).

### 2.2 Structured phantom

**Fig. 1:**
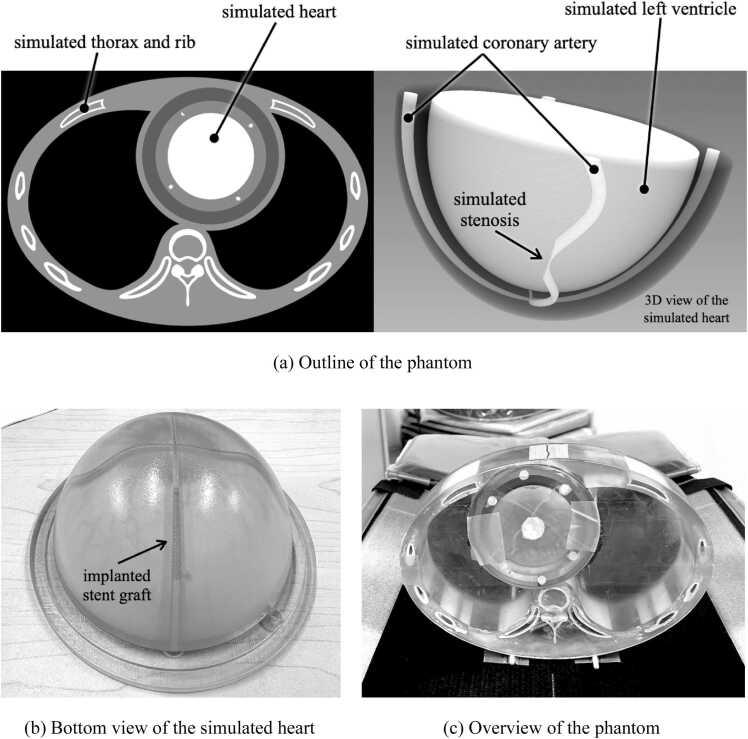
Structured coronary CT angiography phantom.

We have created a phantom for evaluating image quality using a 3D printer (Agilista-3200, KEYENCE, Japan). In order to evaluate image quality under conditions closer to clinical conditions, we created a structured phantom containing structures that simulate the human anatomy, such as vertebrae and ribs. An outline of our phantom is shown in Fig. 1a. The phantom consists of two parts, the thorax, and the heart, with the overall image on the left and a 3D image of the heart part on the right. The base of the phantom was created using a 3D printer, while the bone and vascular cavities were filled with plaster and diluted iodine contrast medium. The outer diameter of the phantom was 30 × 20 cm. Iodixanol was diluted with distilled water to 18 mgI/ml. Simulated coronary arteries with diameters ranging from 4.0 mm to 1.0 mm were created in the heart. Simulated coronary arteries pass through the interior of the simulated myocardium. A 70 % stenotic lesion was created in the simulated coronary artery. A bottom view of an actual photograph of the heart parts is shown in Fig. 1b. We inserted stent grafts (Medtronic BeStent 3.0 × 15 mm, Boston Scientific TAXUS Liberte 2.5 × 28 mm) into the simulated coronary artery. An overall view of the structured phantom is shown in Fig. 1c. The thorax and heart components are combined for CT scanning. The lung region was hollow.

Structured phantoms have digital model data as well as actual objects: digital model data designed for 3D printing have a very high resolution, making them ideal CT images. In this study, the digital model data were considered as the ground truth and compared in image quality with the actual CT images. The digital model data were aligned to the actual CT images and resampled in 1024 matrix size. Because the contrast enhancements of iodine contrast medium depend on the X-ray energy, the CT number of the coronary arteries and left ventricle in the digital model data were determined by measuring the CT images of the actual phantom scans.

### 2.3 Scan protocol and image reconstructions

We scanned the structured phantom with a 320-row detector CT (Aquilion ONE GENESIS, Canon Medical Systems Corporation: CMSC, Japan). The X-ray tube voltage was 120 kV, and the tube current was determined using automatic modulation (noise level 25 HU in images with a thickness of 0.5 mm reconstructed by hybrid iterative reconstruction). The X-ray detector configuration was 0.5 mm × 80 rows, 0.5 s/rot. We scanned the phantom three times repeatedly under helical pitch 0.813.

We reconstructed all images with the following three reconstruction methods, and set the display field of view (D-FoV) to 160 mm centered on the simulated heart. Details of the image reconstruction, including the model data, are listed below.

– hybrid iterative reconstruction: HIR (Adaptive Iterative Dose Reduction 3D: AIDR 3D, CMSC), matrix size: 512 × 512, pixel size: 0.31 × 0.31 mm, slice thickness/interval: 0.5/0.25 mm
– deep learning based reconstruction: DLR (Advanced intelligent Clear-IQ Engine: AiCE, CMSC), matrix size: 512 × 512, pixel size: 0.31 × 0.31 mm, slice thickness/interval: 0.5/0.25 mm
– super-resolution DLR: SR-DLR (Precise IQ Engine: PIQE, CMSC), matrix size: 1024 × 1024, pixel size: 0.16 × 0.16 mm, slice thickness/interval: 0.5/0.25 mm
– model data: matrix size: 1024 × 1024, pixel size: 0.16 × 0.16 mm, slice thickness/interval: 0.25/0.25 mm

### 2.4 Image analysis

We evaluated the quality of CT images reconstructed by the three methods using the indices described below, comparing them with the measured values of the digital model data. All measurements were calculated by averaging the data from three scans. NIH ImageJ software was used for all measurements. We made curved planar reconstruction (CPR) images of simulated coronary arteries using OsiriX MD software.

#### 2.4.1 Image noise and CT number

**Fig. 2:**
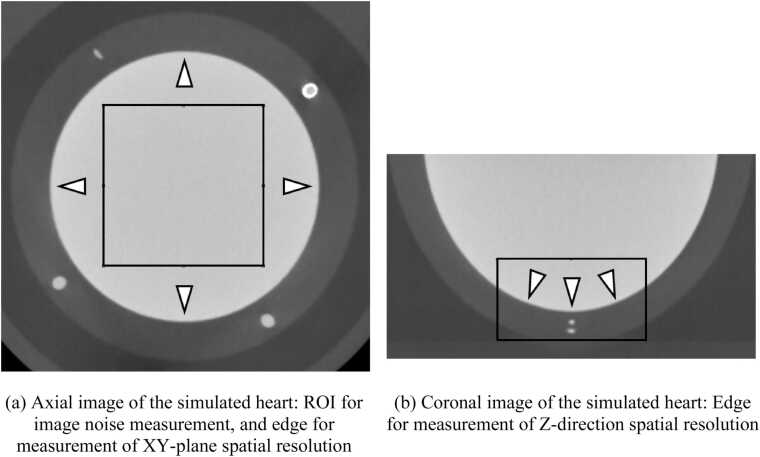
Measurement of image noise and spatial resolution.

We measured the amount of noise by the standard deviation: SD of the CT attenuation numbers and the noise frequency characteristics by the noise power spectrum: NPS, respectively. In addition, CT number of iodine contrast media was also recorded. As shown in Fig. 2a, we measured the noise in a square region of interest: ROI set within the simulated left ventricle. Size of the ROI was 40 × 40 × 25 mm (HIR and DLR: 128 × 128 × 50 voxels, SR-DLR: 256 × 256 × 100 voxels). Each metric was measured slice by slice and averaged.

#### 2.4.2 Spatial resolution

We evaluated the spatial resolution of each image reconstruction method by measuring the task-based MTF [15]. T-MTFs in the XY-plane were measured using the ventricle and myocardial boundaries of the simulated heart (arrows in Fig. 2a). The T-MTF in the Z-direction was measured using the edges within the area indicated by the square in the coronal image shown in Fig. 2b. The edges used for the Z-direction measurements were inclined at a maximum of 30° to the axial plane.

#### 2.4.3 Profile curves

To intuitively evaluate spatial resolution, we analyzed profile curves on CPR images along simulated coronary arteries.

**Fig. 3:**
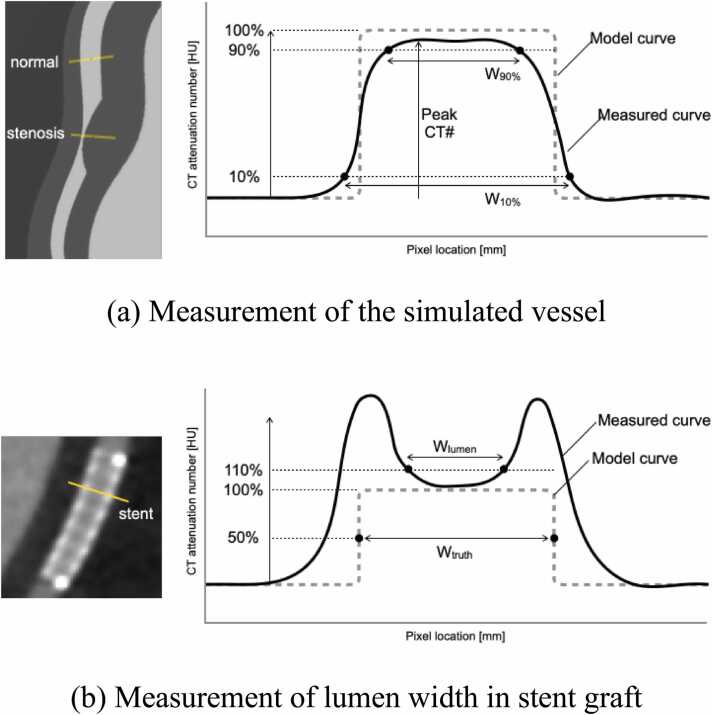
Profile curve analysis.

For the simulated vessels, as shown in Fig. 3a, we measured W_90 %_ and W_10 %_, the widths of the vessels with CT number of 90 % and 10 %, respectively, when the peak CT number of the digital model data was set to 100 %. In addition, we also measured the CT number of the peak. Profile curves were measured at two locations: in a normal vessel and in a vessel with 70 % stenosis.

For the implanted stent graft, the W_lumen_ was measured as shown in Fig. 3b, which is the width of the lumen with a CT number of 110 % when the peak CT number of the digital model data is set to 100 %. In addition, we calculated the error of the W_lumen_ by measuring the vessel width of the digital model data as W_truth_.

## 3 Results

### 3.1 Radiation dose

By the automatic tube current modulation, the X-tube current was adjusted to 120 mA and the CTDIvol was 3.7 mGy. If used in humans, an effective radiation dose would be about 0.83 mSv when scanning an area of 160 mm, assuming a radiation sensitivity factor of 0.014.

### 3.2 Image noise and CT number

Image noise (SD) of the images reconstructed by HIR, DLR, and SR-DLR were 21.1 -, 19.0 -, and 13.1 HU, respectively. The image noise of the SR-DLR was the lowest among all reconstructions. CT number of the images reconstructed by HIR, DLR, and SR-DLR were 422.7-, 422.9-, and 425.3 HU, and average of them (424 HU) was used for CT number of model vessels.

**Fig. 4:**
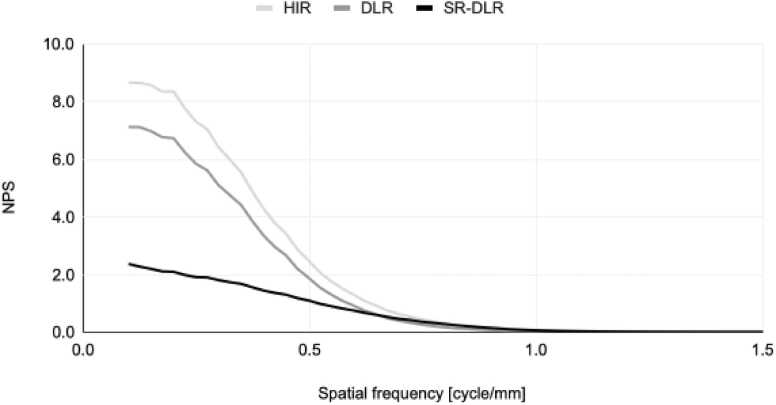
Noise power spectrum (NPS) of each reconstruction.

Fig. 4 shows NPSs of the images reconstructed by HIR, DLR, and SR-DLR. The NPS of the DLR was lower than that of the HIR in all frequency ranges; the NPS of the SD-DLR was drastically lower than both in the low-frequency range and slightly higher in the high-frequency range. Here, the graph is interrupted on the low-frequency side due to the restriction of the ROI size where the NPS was measured. Those values should generally be zero at the origin.

### 3.3 Spatial resolution

**Fig. 5:**
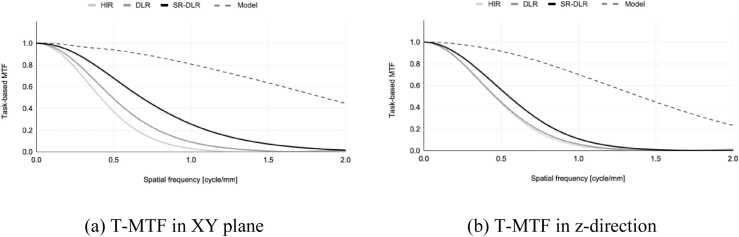
Task-based modulation transfer functions (T-MTF) of each reconstruction.

Fig. 5 shows a comparison of T-MTF, a metric of spatial resolution, for images reconstructed with HIR, DLR, and SR-DLR. Fig. 5a shows the T-MTF in the XY plane measured on the axial image, and Fig. 5b shows the T-MTF in the Z direction measured on the coronal image. The T-MTF of the XY-plane showed a better response for DLR than for HIR and for SR-DLR than for DLR, and the values of spatial frequency at which the T-MTF was 10 % (T-MTF_10 %_) were 0.792, 0.976, and 1.379 cycle/mm, respectively. The T-MTF_10 %_ in the Z direction were 0.849, 0.890, and 1.020 cycle/mm, respectively, with smaller differences compared to those in the XY plane. This may be due to the voxel size being larger in the Z direction than in the XY plane, and the spatial resolution in the Z direction being limited by the Nyquist frequency. For reference, the T-MTF measured on the model data is shown as a dashed line in Fig. 5. The MTF of ideal model data always shows 1.0, whereas the model data used in this experiment was resampled to a matrix size of 1024 × 1024, so the response decayed slowly with increasing spatial frequency.

### 3.4 Profile curves

**Fig. 6:**
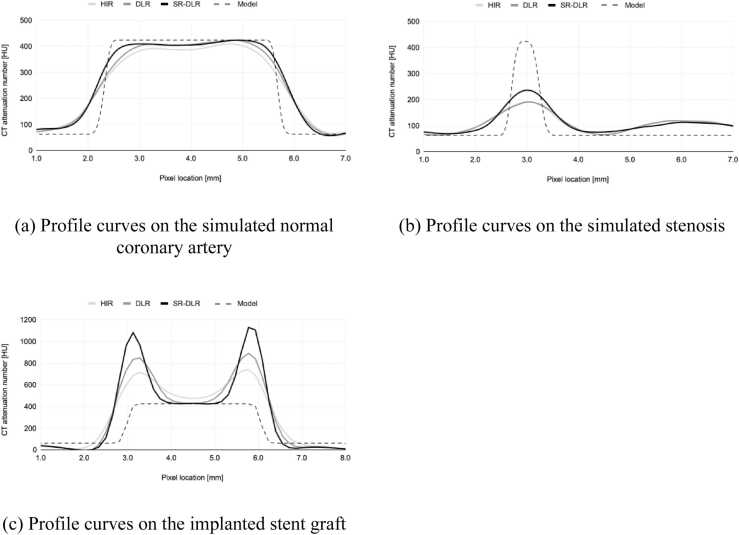
Profile curves of each reconstruction.

Based on MPR images of the simulated coronary arteries, profile curves in the orthogonal direction to the coronary arteries are shown in Fig. 6. Fig. 6a shows a normal coronary artery without stenosis, Fig. 6b shows 70 % stenosis, and Fig. 6c shows an implanted stent graft, comparing the profile curves in the different image reconstruction methods.

Fig. 6a and b show that the SR-DLR profile curves have a shape closest to that of the model data. For the quantitative values Peak CT#, W_10 %_, and W_90 %_, the values of SR-DLR were closest to those of the model data, as shown in Table 1.

**Table 1:** Quantitative values of the profile curve analysis on the simulated coronary artery.

	target	HIR	DLR	SR-DLR	Model
Peak CT# [HU] (error %)	normal artery	409.5 (-3.4 %)	**423.5** **(-0.1 %)**	**423.6** **(-0.1 %)**	424.0 (-)
	stenosis	191.9 (-54.7 %)	190.7 (-55.0 %)	**236.0** **(-44.3 %)**	423.6 (-)
Curve width W_10 %_ [mm] (error %)	normal artery	4.80 (33.5 %)	4.70 (30.8 %)	**4.58** **(27.5 %)**	3.59 (-)
	stenosis	1.75 (124.2 %)	1.82 (133.2 %)	**1.62** **(108.0 %)**	0.78 (-)
Curve width W_90 %_ [mm] (error %)	normal artery	2.08 (-33.9 %)	2.52 (-20.2 %)	**2.91** **(-7.5 %)**	3.15 (-)
	stenosis	0.00 (-)	0.00 (-)	0.00 (-)	0.32 (-)

Bold: The value with the most miniature error.

In the profile curve of the implanted stent graft shown in Fig. 6c, the lumen part of the SR-DLR profile curve was closest to the profile curve of the model without the stent graft (which represents the vessel lumen). Also, CT number on the stent graft was the highest of all on the image reconstructed with SR-DLR. In the quantitative analysis, the lumen width on the image reconstructed with SR-DLR was also closest to the model data shown in Table 2.

**Table 2:** Quantitative values of the profile curve analysis on the implanted stent graft.

	HIR	DLR	SR-DLR	Model (W_truth_)
Curve width lumen [mm] (error %)	0.00 (-)	0.92 (-70.0 %)	**1.40** **(-54.7 %)**	3.08 (-)

Bold: The value with the most miniature error

### 3.5 Representative images

**Fig. 7:**
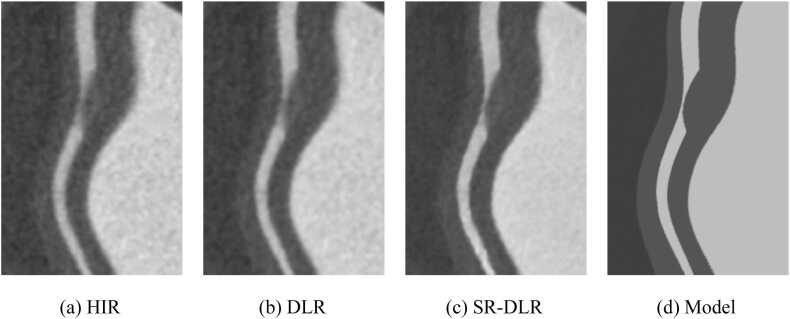
Curved planer reconstructions (CPRs) of the simulated coronary artery with simulated stenosis.

Fig. 7 shows the CPR images of the coronary artery with simulated 70 % stenosis. The stenosis in images reconstructed by HIR and DLR has a partial volume effect that reduces the CT number and makes the lumen less clear, whereas that in SR-DLR is clearly visualized and more similar to the model.

**Fig. 8:**
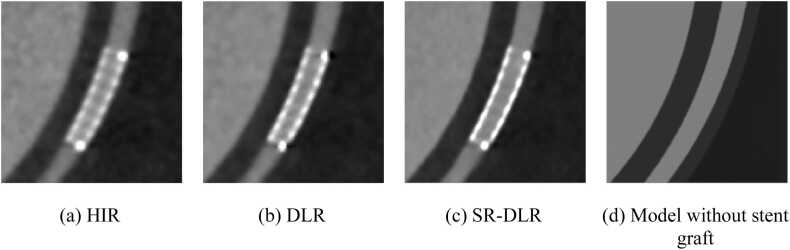
Curved planer reconstructions (CPRs) of the simulated coronary artery with implanted stent graft.

Fig. 8 shows the CPR images of the simulated coronary artery with the implanted stent graft. Only model data images did not have stent graft. The stent graft in the image reconstructed with SR-DLR had higher CT number, better clarity, and less blurring of the boundaries. The lumen of the stent graft was widely delineated and closest to the vessel's width in the model data without the stent graft.

## 4 Discussion

We quantitatively evaluated the performance of SR-DLR using a structured phantom that simulates CCTA. Although it is necessary to scan phantoms to quantitatively evaluate the image quality of reconstruction methods, the performance of nonlinear reconstructions such as DLR depends on the structure of the scanned object [16], [17]. Therefore, it is important to use an object that is similar to the clinical situation in order to accurately evaluate the performance of reconstructions. Previous reports evaluating the image quality of SR-DLR were quantitative evaluations using simple phantoms [12], or qualitative evaluations using clinical images [13], [14], [18], and our study is the first quantitative report using a structured phantom. As the results of quantitative indices measured by structured phantom, SR-DLR had higher spatial resolution and lower noise than either of the conventional reconstruction methods in CCTA.

The noise in the image reconstructed with SR-DLR was lower than with HIR and DLR. The noise distribution of images with previous noise reduction reconstructions tends to be biased towards low frequencies, whereas the frequency distribution of noise in SR-DLR was close to flat. This is also in concordance with previous reports [12]. An increase in the proportion of low-frequency noise results in an oil painting (or plastic) appearance, which is conventionally known to appear in iterative reconstructions, and degrades the texture of the noise [19]. The low-frequency noise was suppressed in the SR-DLR reconstructed images, which made it easier to recognize structures.

The spatial resolution of SR-DLR was superior to other reconstruction methods in both the XY-plane and Z-direction. While general noise reduction methods tend to trade-off between noise reduction and spatial resolution [20], SR-DLR demonstrated high spatial resolution while keeping noise low. The details of the DCNN employed in SR-DLR are undisclosed, but it seems to work as an edge-preserving noise filter. In CCTA, spatial resolution is crucial in all situations, including delicate vascular structures, stenotic lesions, and spotty calcifications. SR-DLR, which improves spatial resolution without the penalty of increased image noise, could contribute to improving the diagnostic accuracy of CCTA examinations.

Profile curves measured from coronary arteries and implanted stent grafts showed the sharpest response in images reconstructed with SR-DLR. This is consistent with the T-MTF measurements and is a natural result. The results of the profile curve measurements are expected to have clinical benefits, e.g., to confirm slight lumen at sites of severe stenosis or to accurately diagnose the lumen of vessels covered by highly absorbent objects, such as stent grafts or severely calcified lesions. This can be visually confirmed from the CPR images of the simulated stenosis shown in Fig. 7 and the inserted stent graft shown in Fig. 8.

The radiation dose in this study was 3.7 mGy in CTDIvol and about 0.83 mSv in effective dose. Although there may be a concern that the tube voltage of 120 kV we used in this experiment may result in higher exposure, this was a clinically acceptable level of radiation dose.

In this study, all noise and spatial resolution indices were measured around the simulated heart. In general, the image characteristics of the image reconstructed with non-linear reconstruction method may be position-dependent, and the results of this study may not indicate the overall characteristic of each reconstruction method. However, as the image FoV and region of interest are limited in CCTA, we consider it acceptable to generalise the data in this study to CCTA. For examination targets such as the trunk, where there is a large variation in organs and region of interests, it may be necessary to measure each index in different positions.

The study has several limitations. The experiments were performed on a phantom, and image quality may vary from actual clinical scans. However, the deviations will be small because we utilized a structural phantom that mimics the human anatomy in this study. In addition, the scan protocol used in this experiment differed from that typically used for CCTA, so the behavior of the SR-DLR may differ from actual clinical scans. However, since the SR-DLR used in this study was based on an image-based DCNN, it is not expected that the behavior of the SR-DLR would vary with the different scan protocols. Another limitation of the study was that it only validated SR-DLRs from a single vendor. Since no other vendors have released SR-DLRs at this time, this will be an issue for future studies. A final limitation of this study was that only experiments at a single radiation dose were performed. If dose reduction protocols will be introduced, the image quality of SR-DLR at various exposure doses will need to be evaluated.

In conclusion, this study validated the image quality of SR-DLR in CCTA. By using a structural phantom that simulates the human anatomy, the experiments were performed under conditions similar to those in actual clinical practice. SR-DLR achieved images with less noise and higher spatial resolution than conventional image reconstructions. SR-DLR can obtain CT images with high spatial resolution and lower noise without the special CT equipment, and will help diagnose CADs in CCTA and other CT examinations that require high spatial resolution.

## Funding statement

Mickaël Ohana and Kazuo Awai received research grant from Canon Medical Systems Corp.

## CRediT authorship contribution statement

**Kazuo Awai:** Writing – review & editing, Supervision. **Ikuo Kawashita:** Validation. **Yuko Nakamura:** Supervision, Conceptualization. **Mickaël Ohana:** Writing – review & editing, Supervision. **Fuminari Tatsugami:** Supervision. **Toru Higaki:** Writing – original draft, Software, Methodology, Investigation, Funding acquisition.

## Declaration of Competing Interest

The authors declare the following financial interests/personal relationships which may be considered as potential competing interests Kazuo Awai reports financial support was provided by Canon Medical Systems Corporation. Mickael Ohana reports financial support was provided by Canon Medical Systems Corporation. If there are other authors, they declare that they have no known competing financial interests or personal relationships that could have appeared to influence the work reported in this paper.
